# Identification of Differentially Expressed Genes and Elucidation of Pathophysiological Relevance of ABCA1 in HaCaT Cells Induced by PM2.5

**DOI:** 10.1155/2021/8862564

**Published:** 2021-04-20

**Authors:** Fen Peng, Chen-Hong Xue, Xiao-Jing Yang, Jing-Yi Huang, Zhou Chen, Jian-Zhong Zhang

**Affiliations:** ^1^Beijing Chao-Yang Hospital, Capital Medical School, Dermatology, Beijing 10020, China; ^2^Peking University People's Hospital, Dermatology, Beijing 100044, China; ^3^Henan Provincial People's Hospital, Dermatology, Beijing 450003, China

## Abstract

**Objective:**

In order to investigate the effects of PM2.5 on proliferation, cell cycle, apoptosis, and potential mechanism of human keratinocyte cell line HaCaT.

**Methods:**

HaCaT cells were treated with different concentrations of PM2.5 suspension for 24 hours. Cell viability was detected by the CCK-8 method. Cell cycle distribution and apoptosis were detected by flow cytometry. Microarray analyses were used to find out the microarray gene expression profiling; data processing included gene enrichment and pathway analysis. Western blot was conducted to validate the key pathways and regulators in the microarray analysis.

**Results:**

The cell activity decreased, and the cell cycle was significantly inhibited with the increase in PM2.5 concentration. Also, by conducting the gene expression microarray assay, we identified 541 upregulated genes and 935 downregulated genes in PM2.5-treated HaCaT cells. Real-time qPCR and western blot confirmed that PM2.5 treatment could induce the expression of ABCA1 while inhibiting that of END1 and CLDN1.

**Conclusion:**

Our results showed that PM2.5 could potentially regulate cell apoptosis and cell cycle arrest via ABCA1-, END1-, ID1-, and CLDN1-mediated pathways in human HaCaT cells, which laid a good foundation for follow-up drug intervention and drug development against skin damage caused by PM2.5 exposure.

## 1. Introduction

The World Health Organization (WHO) reported that air pollution was one of the world's largest environmental health risk factors. Air pollution is a heterogeneous mixture of chemicals and solid particles. Ambient particulate matters (PMs) are one major component of air pollutants. PMs, especially particles on the nanosized range, are the main cause of risk factors [1]. Ambient particulate matter 2.5 (PM2.5) was one of the main components of air pollutants, which can absorb many polycyclic aromatic hydrocarbons and metals [2].

Air pollution-related mortality and morbidity from respiratory and cardiovascular diseases could be traced back to the 1950s. Long-term exposure to PM2.5 is a potential risk factor for various diseases including cancer and cardiovascular and respiratory diseases [3–7]. The skin provides a major defense against air pollutants [8, 9], which can cause human skin damage and exacerbate preexistent skin diseases, such as erythema, hyperplasia, skin aging, atopic dermatitis, and carcinogenesis [10–12]. However, the effects of PM2.5 on the function of human skin and its biological significance in skin homeostasis remain inconclusively understood. Previously, we have demonstrated exposure to PM2.5 is associated with skin damages like senile lentigo [13]. In this study, we focused on the HaCaT cell proliferation and apoptosis under PM2.5 challenge and employed gene microarray analysis to identify upstream regulators and found out that genes associated with an inflammatory response were engaged in PM2.5-stimulated HaCaT apoptosis.

## 2. Methods and Materials

### 2.1. Main Reagents and Equipment

HaCaT cells (Dermatology, Peking University People's Hospital, The second clinical academy of Peking University medicine school), PMI1640 medium (Sigma, USA), trypsin (Gibco, USA), fetal bovine serum (Shanghai Enzyme-linked Biotechnology Co., Ltd.), Rabbit anti-human IL-1 and IL-6 monoclonal antibodies, horseradish peroxidase-labeled goat anti-rabbit IgG (CST, USA), TRIzol reagent, and RT-PCR kit were used (Invitrogen, USA). High-speed refrigerated centrifugation (Beckman, USA), constant temperature CO_2_ incubator, and ABI7900 real-time PCR instrument (ABI, USA) were used.

### 2.2. Methods

#### 2.2.1. Preparation of PM2.5 Turbid Liquid and Particle Treatment

At the junction of the East Second Ring Road and the East Third Ring Road in Beijing, we collected air samples which were 20 m above the top of buildings during the heating period of winter (from December to January). HY-1000 intelligent large-flow TSP sampler (optional PM2.5 cutter, Qingdao Hengyuan Technology Development Co., Ltd.) was used for quartz filter sampling. The average flow rate was set at 1000 L/min, and each sample was continuously collected for 24 hours. The quartz membrane was equilibrated under constant temperature and humidity conditions for 24 hours. Then, the filter was cut into approximately 1 cm^2^ with sterilized surgical scissors and immersed in 75% ethanol, followed by ultrasonic shaking for 60 minutes in a water bath to elute the particles, after which ice was added in to keep the water temperature below 20°C. In the biological safety cabinet, the eluate was filtered into a plurality of glass dishes with 70 *μ*m filters, and the UV lamp was utilized for total sterilization. After most of the ethanol was volatilized, the eluate was vacuum dried for 24 hours, after which samples were weighed. Sterile water was used to prepare a high concentration stock solution which was stored at −20°C. All of the above procedures were processed in the Toxicology Room of the Environmental and Health-Related Product Safety Institute of the Chinese Center for Disease Control and Prevention.

#### 2.2.2. Cell Culture and Cell Counting Kit-8 (CCK-8)

HaCaT cells were cultured in 5% CO_2_ at 37°C in regular Dulbecco's Modified Eagle's Medium (DMEM) (Invitrogen Co. Ltd) containing 1.8 mM Ca^2+^ or with DMEM (Gibco, Life Technologies, Carlsbad, CA, USA) at a low concentration of Ca^2+^ (0.07 mM). Both media were supplemented with 10% heat-inactivated fetal bovine serum, glutamine (2 mM), penicillin (100 U/ml) (Euroclone), and streptomycin (100 mg/ml) (Euroclone). Cells were stimulated with different concentrations of PM2.5 (50 *μ*g/ml, 100 *μ*g/ml, 200 *μ*g/ml, and 400 *μ*g/ml) in HaCaT cells at 24 h and 48 h. Cells were seeded in 96-well plates at a density of 2.0 × 10^3^ cells per well. Each sample was repeated five times. Then, cell proliferation was determined using CCK-8 (Sigma-Aldrich, St. Louis, MO, USA) for five days after seeding. In brief, CCK-8 solution was added to each well and incubated at 37°C for 4 h according to the manufacturer's protocol. Following this, the optical density (OD) value at 450 nm was determined using a microplate reader (Tecan Infinite, Männedorf, Switzerland).

#### 2.2.3. Microarray Analysis

HaCaT cells were treated by 200 ug/ml PM2.5, and untreated HaCaT cells were collected for microarray analysis, microarray gene expression profiling, and data processing.

(1) *RNA Preparation and Quality Control*. Total RNA from cultured HaCaT cells was extracted by TRIzol and purified using RNeasy RNA extraction kit. Quality control of extracted RNA was subsequently validated by both Thermo Nanodrop 3000 and Agilent 2100 bioanalyzer with utilization of Agilent RNA 6000 Nano Kit. Total RNA will be subjected to microarray analysis until it meets the following standards: 1.8 <A260/A280 <2.0 by Thermo Nanodrop 3000 and RIN> = 7.0 and 28S/18S >0.75 by Agilent 2100 bioanalyzer.

*(2) Microarray Processing and Data Analysis*. 6 GeneChip microarrays (Affymetrix 901838) were hybridized with three pairs of samples to determine gene expression profiles of the control and treatment samples according to the manufacturer's instructions. Finally, raw data were imported to R (http://www.r-project.org) and analyzed by the Bioconductor affy package (http://www.bioconductor.org). Logarithmic (base 2) intensity measures were obtained by RMA. The intensity was converted to nonlogarithmic values and rescaled by adjusting mean intensity on each array to 400. Cell files and RMA values were deposited on Gene Expression Omnibus (http://www.ncibi.nih.gov/geo/).

*(3) Identification of Differential Expressed Genes (DEGs)*. Limma package was used to normalize the microarray raw data, and genes with (log2fold change) > = 2 and *P* < 0.05 indicated that there is a statistically significant difference between the groups.

*(4) Enrichment Analysis of DEGs*. The online functional annotation tool, DAVID (http://www.abcc.ncifcrf.gov), was then used to perform the GO-BP functional enrichment analysis for DEGs, with the threshold of *P* < 0.01. Pathway enrichment analysis was done using both KEGG (http://www.kegg.jp/Kegg/pathway.html) and Reactome (http://www.reactome.org) databases. *P* < 0.01 was selected as the threshold value.

*(5) Pathway and Network Analysis*. The list of significantly overexpressed or downregulated genes identified by Affymetrix probe set IDs, fold changes, and *p* values were uploaded into the Ingenuity Pathway Analysis (IPA) tool (http://www.ingenuity.com). Each clone identifier was mapped to its corresponding gene object in the Ingenuity Pathway Knowledge Base (IPKB). These focus genes were then used for constructing biological networks, using the “IPA” core analysis function. To start building networks, the application queries the IPKB for interactions between focus genes and all other gene objects stored in the knowledge base and generates a set of networks. Every resulting gene interaction has supporting literature findings available online. IPA then computes a score for each network according to the fit of the user's set of significant genes. The score is derived from *P* value and indicates the likelihood of the focus genes in a network being found together as a result of random chance. A score of 2 indicates that there is a 1-in-100 chance that the focus genes are together in a network as a result of random chance. Therefore, scores of 2 or higher have at least 99.5% confidence of not being generated by random chance alone.

#### 2.2.4. Western Blot

Total protein was extracted from cells and quantified. Transmembrane was performed after electrophoresis. After blocking with 5% skim milk for 1.5 h, membranes were incubated with primary rabbit anti-human monoclonal antibodies of IL-1, IL-6, and GAPDH (1 : 1000; Cat nos: #12703, #12153, #8884; Cell Signaling Technology, Danvers, MA, USA) overnight at 4°C. After washing with TBST three times, 5 min each time, membranes were incubated with Horseradish peroxidase-labeled goat anti-rabbit IgG secondary polyclonal antibody (1 : 2000; Cat nos: A0208, Beyotime Biotechnology, Shanghai, China) at 37°C for 2 h. After washing with TBST three times, 10–15 min each time, color development was performed, and the signal was detected. GAPDH was used as endogenous control.

#### 2.2.5. Real-Time PCR

PCR reactions were performed using real-time PCR kit with a reaction system of 10 *μ*l. PCR reaction conditions were as follows: 95°C for 10 min, followed by 35 cycles of 95°C for 30 s, 59°C for 30 s, and 72°C for 25 s. In this study, Roche 384 real-time PCR amplification instrument was used to carry out real-time fluorescence quantitative PCR reactions with GAPDH as endogenous control. Data were processed using the ΔΔCt method, and three replicates were set for each sample.

#### 2.2.6. Flow Cytometry

Cell cycle and apoptosis were determined using flow cytometry. In brief, cells were seeded in 6 cm dish overnight to a confluency of 80%. Then, cells were trypsinized, washed with precooled D-Hanks (pH = 7.2–7.4) buffer, and fixed with 75% ethanol at 4°C for 1 h. Following this, cells were stained with propidium iodide (PI) solution (PI 50 *μ*g/mL and RNase A 200 *μ*g/mL, Sigma-Aldrich, St. Louis, MO, USA) at RT for 30 min in the dark and then analyzed using a Guava®easyCyte flow cytometer (Millipore, Billerica, MA, USA). Each experiment was performed in triplicate, and the average value was calculated as the final result.

## 3. Results

### 3.1. Effect of PM2.5 on Cell Viability, Cell Cycle, and Cytokine Secretion

**Figure 1:**
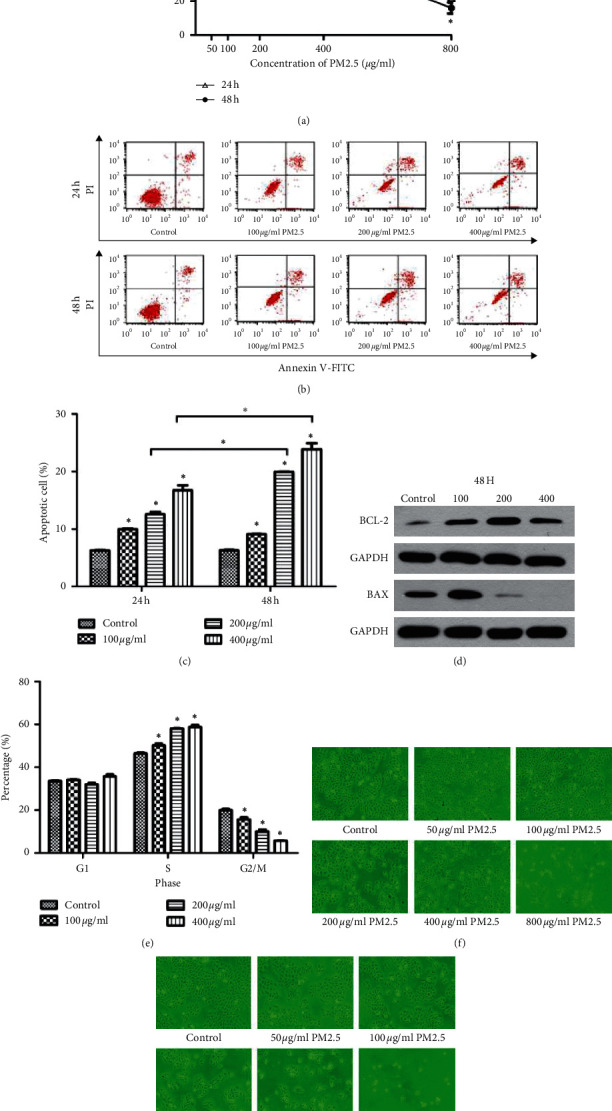
(a) Stimulated with different concentrations of PM2.5 (50 ug/ml, 100 ug/ml, 200 ug/ml, and 400 ug/ml) in HaCaT cells at 24 h and 48 h, the cell activity decreased with the increase in PM2.5 concentration. (b–e) Stimulated with different concentrations of PM2.5 (50 ug/ml, 100 ug/ml, 200 ug/ml, and 400 ug/ml) in HaCaT cells at 24 h and 48 h, the cell cycle was significantly inhibited with the increase in PM2.5 concentration. (f, g) HaCaT cells after 24 h and 48 h PM2.5 treatment observed under microscope. ^*∗*^*P* < 0.05.

Stimulated with different concentrations of PM2.5 (50 ug/ml, 100 ug/ml, 200 ug/ml, and 400 ug/ml) in HaCaT cells at 24 h and 48 h, the cell activity decreased with the increase in PM2.5 concentration (Figure 1(a)). Stimulated with different concentrations of PM2.5 (50 ug/ml, 100 ug/ml, 200 ug/ml, and 400 ug/ml) in HaCaT cells at 24 h and 48 h, the cell cycle was significantly inhibited with the increase in PM2.5 concentration (Figures 1(b) and 1(c)). Cell apoptosis HaCaT cells after 24 h and 48 h PM2.5 treatment were observed under microscope (Figures 1(d) and 1(e)).

### 3.2. Identify Differentially Expressed Genes Stimulated by PM2.5 on HaCaT Cells

**Figure 2:**
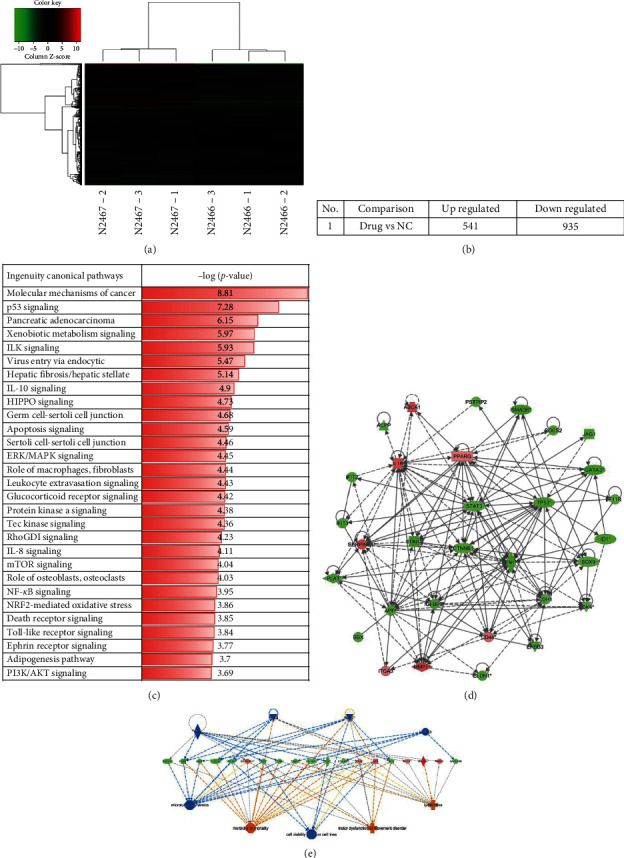
(a, b) 541 of 1476 DEGs were upregulated in HaCaT cells after PM2.5 stimuli, while 935 genes were downregulated. (c) Ingenuity canonical pathway analysis indicated inflammatory response-associated pathways are deregulated in PM2.5-treated HaCaT cells, like IL-10, IL-8, and NF-*κ*B signaling. The differential genes of different treatment groups were selected by the IPA grid algorithm to select genes with noticeable downstream changes. (d) Gene network, showing the interaction between different treatment groups through the grid diagram relationship. (e) The regulatory effect network map shows the interaction between genes and regulators and functions in the dataset.

To decipher the mechanism underlying PM2.5-induced HaCaT cell cycle arrest and apoptosis, we performed gene expression microarray analysis and identified 1476 differentially expressed genes (DEGs) between 200 *μ*g/ml PM2.5-treated HaCaT cells and control cells. 541 of 1476 DEGs were upregulated in HaCaT cells after PM2.5 stimuli, while 935 genes were downregulated (Figures 2(a) and 2(b)). Ingenuity canonical pathways analysis indicated inflammatory response-associated pathways are deregulated in PM2.5-treated HaCaT cells, like IL-10, IL-8, and NF-*κ*B signaling (Figure 2(c)). The differential genes of different treatment groups were selected by the IPA grid algorithm to select genes with obvious downstream changes (ABCA1, ACPP, BBX, CD44, CDH1, CDK4, CLDN1, CTNNB1, EDN1, ERBB3, FN1, F11R, GATA3, ID1, IFIT2, IFIT3, IGFBP3, IL1B, ITGA3, JAG1, MMP1, PLAT, PPARG, PSTPIP2, SERPINE1, SMAD6, SOCS2, sox9, STAT3, and TP53) and map the gene network, showing the interaction between different treatment groups through the grid diagram relationship (Figure 2(d)). The regulatory effect network map shows the interaction between genes and regulators and functions in the dataset. The first regulatory network in this regulation effect analysis shows that the dataset may be due to the regulation of PLK2, PLK4, TAZ, and VGLL3 through ADM, BASP1, CD44, CDH2, CITED2, COL12A1, CYP1B1, CYR61, EDN1, FN1, GADD45B, IFIT2, IL1B, MYOF, NAV3, PLAT, SERPINE1, SOX9, and THBS1 and other genes have an activating effect on lymphoma, morbidity or mortality, motor dysfunction, or movement disorder, inhibiting cell viability of tumor cell lines and microtubule dynamics (Figure 2(e)).

### 3.3. Validate DEGs between PM2.5-Treated and Control HaCaT Cells

To determine the critical regulators in 200 *μ*g/ml PM2.5-treated HaCaT cells, we performed RT-qPCR to validate the differentially expressed genes. After being treated with 200 *μ*g/ml PM2.5, HaCaT cells enriched expression of ITGA3, ABCA1, CD44, IL-1*β*, MMP1, and SERPINE1 (Figure 2(a)) while downregulated expression of EDN1 and ID1 (Figure 2(b)).

### 3.4. Effects of PM2.5 on the Expression of DEGs by Western Blot

**Figure 3:**
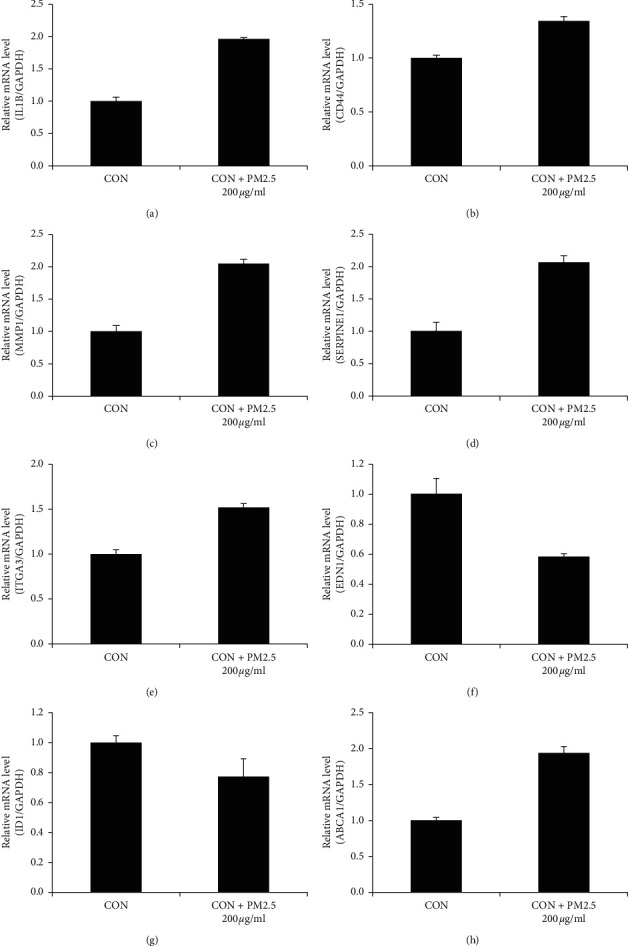
HaCaT cells enriched expression of ITGA3, ABCA1, CD44, IL-1*β*, MMP1, and SERPINE1 while downregulated expression of EDN1 and ID1.

We further confirmed the expression of DEGs at protein level by western blot. It was found that the relative expression level of ABCA1 was significantly higher in HaCaT cells stimulated with 200 *μ*g/ml PM2.5 for 24 h and that of ID1 was significantly higher in the control group (Figure 3). However, no significant difference remained in the expression of END1, CLDN1, TP53, NRF2, and IL-1*β* between PM2.5-stimulated HaCaT cells and control group (Figure 3).

## 4. Discussion

**Figure 4:**
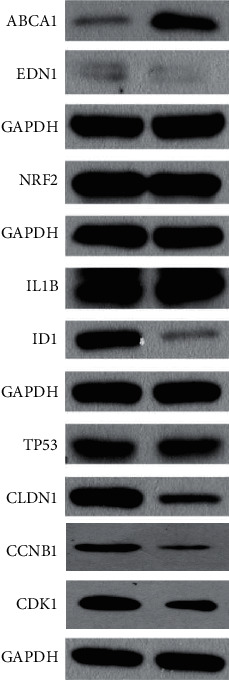
The relative expression level of ABCA1 was significantly higher in HaCaT cells stimulated with 200 *μ*g/ml PM2.5 for 24 h and that of ID1 was significantly higher in control group (Figure 4). However, no significant difference remained in the expression of END1, CLDN1, TP53, NRF2, and IL-1*β* between PM2.5-stimulated HaCaT cells and control group.

It has been widely documented that PM2.5 contributes to the development of respiratory diseases [14]. However, its effects on human skin have not been fully elucidated. It is generally accepted that there are two potential pathways for PM2.5 to penetrate the skin surface: (1) via hair follicles or sweat ducts and (2) across the stratum corneum [15]. It is believed that particles may penetrate barrier-disrupted skin [15, 16]. Even in barrier-intact skin, PM2.5 can penetrate almost every follicle (Figure 1). Moreover, PM2.5 is reported to degrade the skin barrier by reducing the levels of cytokeratin, filaggrin, E-cadherin, and tight junction molecules [12, 17]. In barrier-disrupted skin, PM2.5 can penetrate into the dermal layer after repeated application [15]. However, its biological significance in skin homeostasis is not fully understood (Figure 4).

Because of its high surface area, PM2.5 contains environmental metals and PAHs [18]. PAHs can activate aryl hydrocarbon receptor (AhR), while metals may generate ROS by Fenton-like reactions [19–21]. Upregulation of Inflammatory cytokines as a consequence of increased cellular oxidative stress. Previous study indicated that treatment with PM2.5 upregulates the levels of IL-6, IL-1*α*, and TNF-*α* mRNA production in the keratinocytes [22]. However, the accurate effects of PM2.5 on the function of human skin remain inconclusively understood.

Long-term high concentration of PM2.5 can cause toxicity to cells and cause oxidative stress, DNA damage, mutation, and even cancer, while low concentration of PM2.5 can cause abnormal expression of various inflammatory-related genes [1, 23–25]. It is necessary to further explore the changes in genetic information and pathways of cells stimulated by PM2.5. We collected PM2.5 in the heating season of Beijing from 2015 to 2016 and prepared a suspension to stimulate HaCaT cells. In the subsequent experiments, in order to prove that, even under given lower dosages, we could still detect significant effects of PM2.5, and we used 200 ug/ml PM2.5 to treat HaCaT cells and then performed transcriptome analysis on cells not treated with PM2.5. We found a number of genes such as DEGS, ITGA3, ABCA1, CD44, IL-1*β*, MMP1, ACPP, PSTPIP2, GATA3, SMAD6, EDN1, ID1, IGFBP3, and PLAT with statistically significant differential expression. These genes involved are in inflammation and related to pathways such as oxidative stress [26]. PM2.5 may affect the function of cells by affecting the expression of other genes, among which ABCA1 was to be closed related to PM2.5. The ATP-binding cassette A1 (ABCA1) is mainly discussed as the rate-limiting step in the biogenesis of high-density lipoproteins (HDLs) [27]. The role of ABCA1 in other systems has not been reported yet. Actually, ABCA1 was reported to be an activator in multiple cell pathways such as cell inflammation and macrophage foam cell formation [28]. Endothelin 1 gene (EDN1) is one of the families of three endothelins that exert their action through two G-protein-coupled receptors [29]. As the prominent endothelial mediator, EDN1 had a vasoconstrictive action and could induce cell proliferation [30], while claudin 1 (CLDN1) is associated with risk of several cancers [30], indicating that PM2.5 may affect vessels and cancer, but the mechanisms between these genes and skin health need further exploration.

According to the difference in expression of each gene verified by transcriptome chip and western blot, it is clear that long-term exposure of skin to PM2.5 is easy to produce differential expression of various genes under its action. Abnormal expression of different genes makes the microenvironment of cells change, promotes cell-mediated inflammation changes, and further promotes cell cancer. Simultaneously, its secretion of cytokines and inflammatory factors to the periphery may affect the surrounding organs and tissues due to epidermal cell damage. Defining changes in each gene lays a good foundation for follow-up drug intervention and drug development. Further application of this study will provide strategies for fighting against skin damage caused by PM2.5 exposure.

## Data Availability

All data generated or analyzed during this study are included within this article.

## Supplementary Materials

Supplementary Materials Supplementary Figure 1S. There is no significant change in cell cycle after PM2.5 treatment.
