# Supplementary material for: Identification of Differentially Expressed Genes and Elucidation of Pathophysiological Relevance of ABCA1 in HaCaT Cells Induced by PM2.5
Source: Bioinorg Chem Appl. 2021 Apr 20;2021:8862564. doi: 10.1155/2021/8862564 (PMC8079182; doi:10.1155/2021/8862564)
Supplement: Supplementary Materials — Supplementary Figure 1S. There is no significant change in cell cycle after PM2.5 treatment. [file 8862564.f1.docx]

Supplementary figure S1


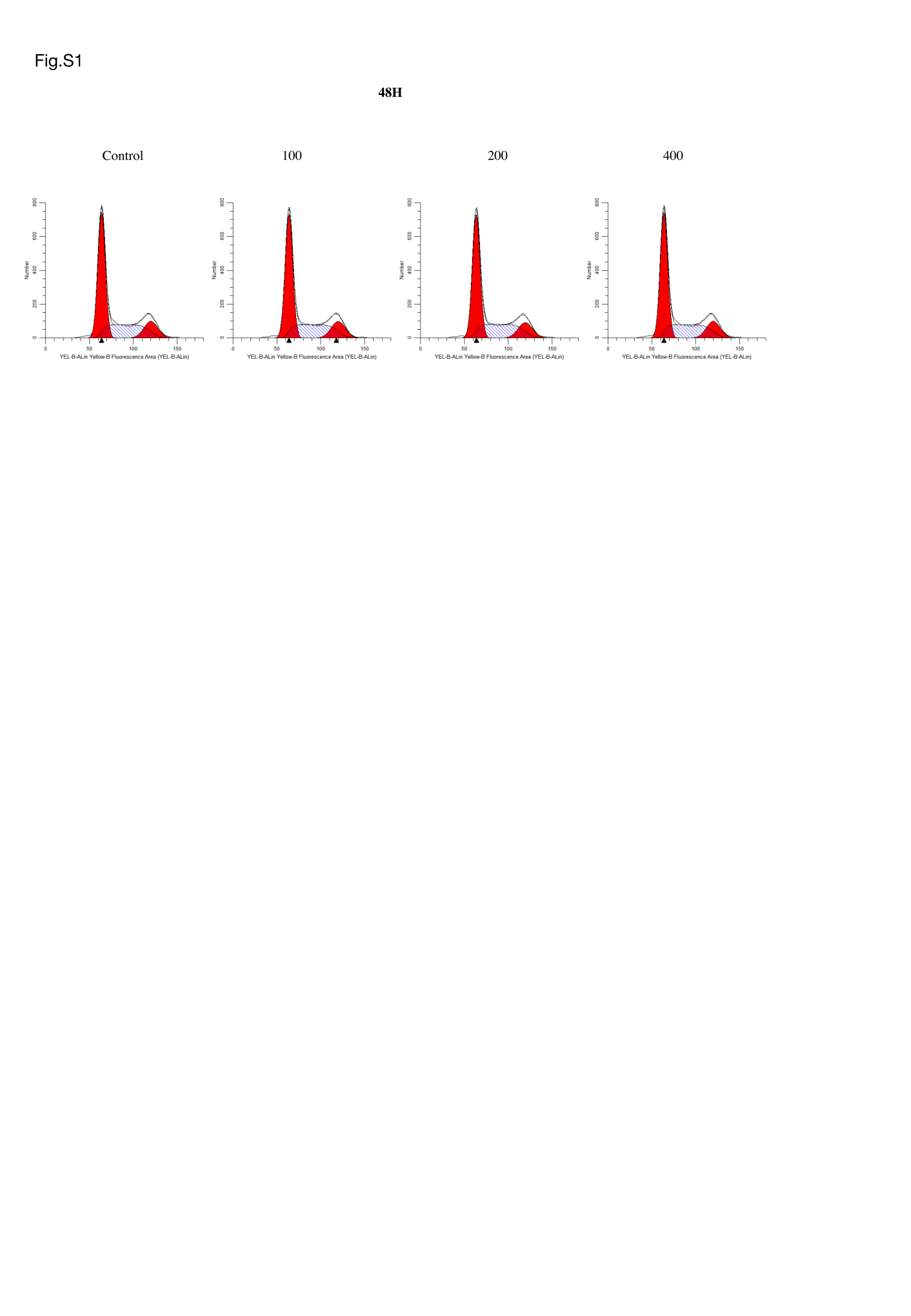


Supplementary figure 1S. There is no significant change in cell cycle after PM2.5 treatment.
